# Pilot Study on Analysis of Electroencephalography Signals from Children with FASD with the Implementation of Naive Bayesian Classifiers

**DOI:** 10.3390/s22010103

**Published:** 2021-12-24

**Authors:** Katarzyna Anna Dyląg, Wiktoria Wieczorek, Waldemar Bauer, Piotr Walecki, Bozena Bando, Radek Martinek, Aleksandra Kawala-Sterniuk

**Affiliations:** 1 St. Louis Children Hospital, 31-503 Krakow, Poland; katarzyna.dylag@dzieciecyszpital.pl (K.A.D.); krasnoludki11a@poczta.onet.pl (B.B.); 2 Department of Pathophysiology, Jagiellonian University in Krakow—Collegium Medicum, 31-121 Krakow, Poland; 3 Department of Bioinformatics and Telemedicine, Jagiellonian University in Krakow—Collegium Medicum, 30-688 Krakow, Poland; wiktoria.wieczorek@student.uj.edu.pl (W.W.); piotr.walecki@uj.edu.pl (P.W.); 4 Department of Automatic Control and Robotics, AGH University of Science and Technology, 30-059 Krakow, Poland; 5 Department of Cybernetics and Biomedical Engineering, VSB—Technical University Ostrava—FEECS, 708 00 Ostrava-Poruba, Czech Republic; radek.martinek@vsb.cz; 6 Faculty of Electrical Engineering, Opole University of Technology, 45-758 Opole, Poland

**Keywords:** digital signal processing, electroencephalography (EEG), Naive Bayesian classifiers, Fetal Alcohol Spectrum Disorders (FASD)

## Abstract

In this paper Naive Bayesian classifiers were applied for the purpose of differentiation between the EEG signals recorded from children with Fetal Alcohol Syndrome Disorders (FASD) and healthy ones. This work also provides a brief introduction to the FASD itself, explaining the social, economic and genetic reasons for the FASD occurrence. The obtained results were good and promising and indicate that EEG recordings can be a helpful tool for potential diagnostics of FASDs children affected with it, in particular those with invisible physical signs of these spectrum disorders.

## 1. Introduction

Fetal Alcohol Spectrum Disorders (FASD) is an umbrella term used to describe the spectrum of conditions resulting from prenatal alcohol exposure (PAE) [1,2,3].

The negative consequences of maternal alcohol consumption on the fetus were first described in the medical literature in the 1973 by Jones and Smith [4,5], who pointed at dysmorphic features and developmental problems noticeable in children born from mothers with the history of alcohol abuse and created a term: fetal alcohol syndrome (FAS). Further studies have determined that the impaired function of the central nervous system, not the presence of facial dysmorphism was the characteristic of the symptomatic patients with PAE and the term FASD was created to encompass a broader spectrum of conditions [6,7]. The FASD is characterized by a high worldwide prevalence [8,9] and is one of the leading causes of developmental disability in developed countries and remains one of the very few preventable ones [10]. Thus, only in the United Stated the prevalence is estimated to be from 1.1% to 5.0. In Poland the prevalence of FASD exceeds 2% [3,11,12].

Regardless the high prevalence of diagnosis of FASD remains a challenge. The lack of a specific biomarker [13,14] or a single imaging study [15] that could confirm diagnosis leads do the fact that diagnosis is currently solely based on the clinical criteria [16,17,18,19,20,21,22]. In the absence of characteristic dysmorphic features, the diagnosis of FASD can be made only when PAE is confirmed [17,18,19,20,21]. However, an incomplete medical history and the loss of contact with the birth mother often lead to missed diagnoses. A pathophysiological mechanism in which PAE affects the central nervous system (CNS) is not fully understood [2,23] yet the neurons function is undoubtedly impaired [24]. The influence of PAE on the electrophysiological activity of the brain and a high incidence of both epilepsy and single episode of clinical seizures were established by Bell at al. [25]. Abnormal brain activity associated with the PAE has even been observed in neonates [26].

A few studies, have investigated the characteristics of EEG of children and adolescents with FASD [27,28,29], but these analyses were limited to only qualitative, clinical aspects. Interdisciplinary diagnostic tools include classifiers, which are trained in order to determine a class of an unknown feature and are used for results prediction, pattern recognition and classification. Classification techniques can be divided into two types [3,30]: Binary classification: (two classes, e.g., negative or positive); Multi-classification (multiple classes, e.g., diagnostic or prognostic score). There are also other naive Bayesian classifiers.

The use of Naive Bayesian classifiers for various assessments or diagnostics purposes is not the newest method, but its use can provide some promising results and is being applied for various pattern-recognition related purposes. In [31] they were applied for lower limb movement detection (based on EEG) for the purpose of potential lower limb prosthesis control. In [32] the authors applied among the others Bayesian classifiers for the purpose of autism diagnostics with the efficiency of 65–76%, which is a good results in such studies.

Bayesian statistics are a very useful tool, suitable for various diagnostics supporting applications. An interesting approach regarding FASD detection using a Bayesian framework was presented in [2], which is only one of very few studies regarding FASD, where Bayesian classifiers were applied. Zhang et al. [3] presented their FASD detection methodology based on various biomedical sources, such as eye-movement analysis, psycho-metrics and neuroimaging processing data, which made their solution more reliable and versatile. A similar study, where various sub-tests (three eye-movement- related and three psychometric tests,, but with no EEG analysis) was described in [7]. An overall accuracy of 52.2% with the use of naive Bayes, and an overall accuracy of 85% was achieved with the use of various Machine Learning-based methods.

We applied naive Bayesian classifiers in this study for the analysis of EEG data in order to diagnose FASD. The obtained results are promising; however, further amendments, improvements and investigations are still required. A thorough literature study did not provide any information regarding using naive Bayesian classifiers for the FASD assessment in children and/or adolescents based on EEG data analysis. In our opinion, using EEG is safe, inexpensive and may provide useful information, which cannot be detected using other screening tool, such as those based on eye-movement or psychometrics [33,34].

In this work we propose a novel classical naive Bayesian classifiers-based method for the purpose of FASD diagnostics using electroencephalography signals. Based on a thorough literature study, similar methods have not been applied for the assessment of FASD.

## 2. Materials and Methods

### 2.1. Study Participants

EEG recordings were obtained from a database of Diagnostics Center, St. Louis Hospital in Krakow (Poland). The studies were carried out in the years 2013–2020. The study group consisted of 50 children aged 7–13 (22 males, 28 females), who received a diagnosis of FASD according to the criteria published by Hoyme et al. [6,19]. The patients had EEG performed as a part of routine neuropsychiatric evaluation. Additionally data from a control group (50 participants, also aged 7–13, 15 males, 35 females) consisting of patients from the Pediatric Neurology, Rheumatology and General Pediatrics Department of the same hospital, who had undergone EEG as a part of evaluation due to syncope or cyclic vomiting or abdominal migraine, was also analyzed.

The Exclusion criteria for both study groups were:

- Diagnosed epilepsy and/or treatment with anti-epileptic medications;
- Other neurological disease that can affect the bioelectrical activity of the brain (neurometabolic diseases, known structural defects of the central nervous system or other CNS defects);
- Systemic conditions that could temporarily affect the EEG signal abnormalities in the general condition at the time of the test (active infectious disease);
- High body temperature (fever, dehydration).

On 12 February 2021, the Bioethics Committee of the Regional Medical Chamber in Krakow approved the described retrospective study (approval no. 12/KBL/OIL/2021).

### 2.2. Experimental Setup

Recordings of the electroencephalographic signals were made with the use of a 32-channel EEG apparatus (Elmiko). The mean EEG recording duration in one patient was approximately 15 min. The registration took place under the following conditions:

- Resting activity, in which the patient stayed for a specified time with eyes open and then with the eyes closed;
- Activation tests, in which photo-stimulation was used with flashing lights of various frequency ranges (2–30 Hz) for 2 min 30 s together with hyperventilation, which required patient’s cooperation, i.e., taking slow, deep breathing for about 3 min.

Signals were recorded with the sampling frequency $F_{s}=250$ Hz. In order to remove the electrical interference of 50 Hz a notch filter was applied. The electrodes were placed in accordance with the double-banana system, instead of the classic “10–20” arrangement, which is a typical clinical montage used usually in epilepsy-related studies [34,35,36].

Signals recorded with the electrodes (18 channels) placed in the following locations: ’Fp2-F8’, ’F8-T4’, ’T4-T6’, ’T6-O2’, ’Fp1-F7’, ’F7-T3’, ’T3-T5’, ’T5-O1’, ’Fp2-F4’, ’F4-C4’, ’C4-P4’, ’P4-O2’, ’Fp1-F3’, ’F3-C3’, ’C3-P3’, ’P3-O1’, ’Fz-Cz’, ’Cz-Pz’.

### 2.3. Data Analysis

For the analysis, we decided to apply a naive Bayesian classifier. EEG frequency wave data from each channel were filtered to obtain **’alpha’**, **’beta’**, **’gamma**, **’delta’** and **’theta’** signals. Then, from the filtered signals, the characteristics were calculated in the form of the following parameters:

- Root-mean-square level,
- Skewness,
- Kurtosis,
- Peak-magnitude-to-RMS ratio,
- Peak to peak,
- Power of lower and high envelop,
- Power of the signal,
- Minimum and maximum value of the signal.

All calculated signal characteristics were collected into vectors and then combined with each other. In this way, a single vector describing a specific study was obtained. The last step was to assign labels to vectors describing their belonging to the control class or to the sick. The naive Bayesian classifier was trained on this vector.

Naive Bayes classifier is a very popular method for classification and categorisation, as it applied the Bayes theorem in order to separate particular data based on simply trained features. It requires only a small number of training data set, which is its high advantage [33].

The Naive Bayes algorithm is a classification technique based on Bayes’ Theorem with an assumption of independence among predictors. In simple terms, a naive Bayes classifier assumes that the presence of a particular feature in a class is unrelated to the presence of any other feature. The Bayes theorem provides a way of calculating posterior probability P(c|x) from P(c), P(x) and P(x|c) as in the Equation (1) below:

(1) $$P\left(c|x_{1},\cdot ,x_{n}\right)=\frac{P\left(x_{1},\cdot ,x_{n}|c\right)P\left(c\right)}{P(x_{1},\cdot ,x_{n}),}$$

where:

- $x_{i}$ are the described attributes;
- *c* are classes;
- $P(c|x_{1},\cdot ,x_{n})$ is the posterior probability of class *c* given predictor *x*;
- P(c) is the prior probability of that class;
- $P(x_{1},\cdot ,x_{n}|c)$ is the likelihood which is the probability of the predictor for the given class;
- $P(x_{1},\cdot ,x_{n})$ is the prior probability of the predictor.

Using assumptions about the naive condition independence we can state that (see (2)):

(2) $$P\left(x_{i}|y,x_{1},\cdot ,x_{i-1},x_{i+1}\cdot x_{n}\right)=P\left(x_{i}|y\right).$$

And for all *i* we can formulate the below Equation (3):

(3) $$P\left(c|x_{1},\cdot ,x_{n}\right)=\frac{P\left(c\right)\prod_{i=1}^{n}P\left(x_{1},\cdot ,x_{n}|c\right)}{P(x_{1},\cdot ,x_{n})}.$$

Since the $P(x_{1},\cdot ,x_{n})$ is constant and regardless of the classes it can be expressed with the following approximation (4):

(4) $$P\left(c|x_{1},\cdot ,x_{n}\right)\approx P\left(c\right)\prod_{i=1}^{n}P\left(x_{1},\cdot ,x_{n}|c\right).$$

Using the above dependence, it is possible to determine class membership using the following formula (5):

(5) $$\hat{c}=\underset{c}{\mathrm{argmax}}P\left(c\right)\prod_{i=1}^{n}P\left(x_{1},\cdot ,x_{n}|c\right).$$

## 3. Results

**Figure 1:**
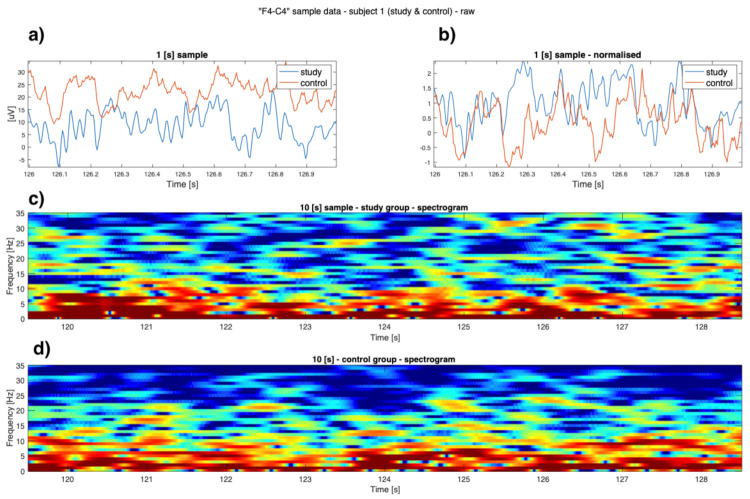
Example raw data from channel **’F4-C4’**: (**a**) time-series of both study group and control group (1 [s]); (**b**) normalised time series of study group and control group (1 [s]); (**c**) 10 [s] sample of FASD (top spectrogram); (**d**) 10 [s] of healthy control (bottom spectrogram)—sample 1.

**Figure 2:**
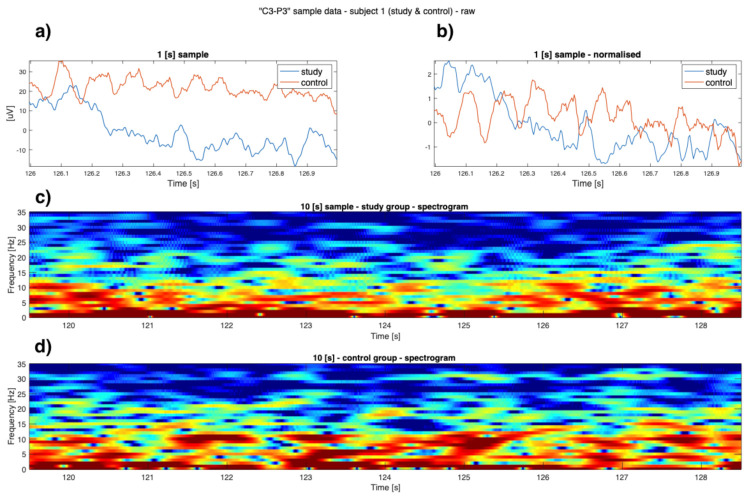
Example raw data from channel **’C3-P3’**: (**a**) time-series of both study group and control group (1 [s]); (**b**) normalised time series of study group and control group (1 [s]); (**c**) 10 [s] sample of FASD (top spectrogram); (**d**) 10 [s] of healthy control (bottom spectrogram)—sample 1.

**Figure 3:**
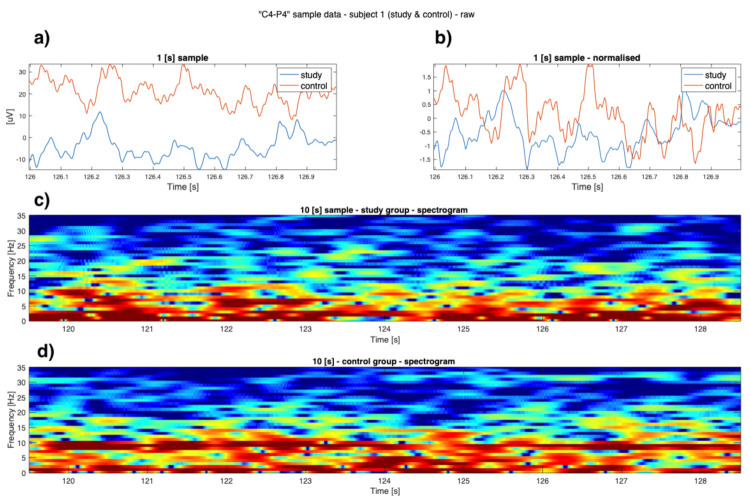
Example raw data from channel **’C4-P4’**: (**a**) time-series of both study group and control group (1 [s]); (**b**) normalised time series of study group and control group (1 [s]); (**c**) 10 [s] sample of FASD (top spectrogram); (**d**) 10 [s] of healthy control (bottom spectrogram)—sample 1.

**Figure 4:**
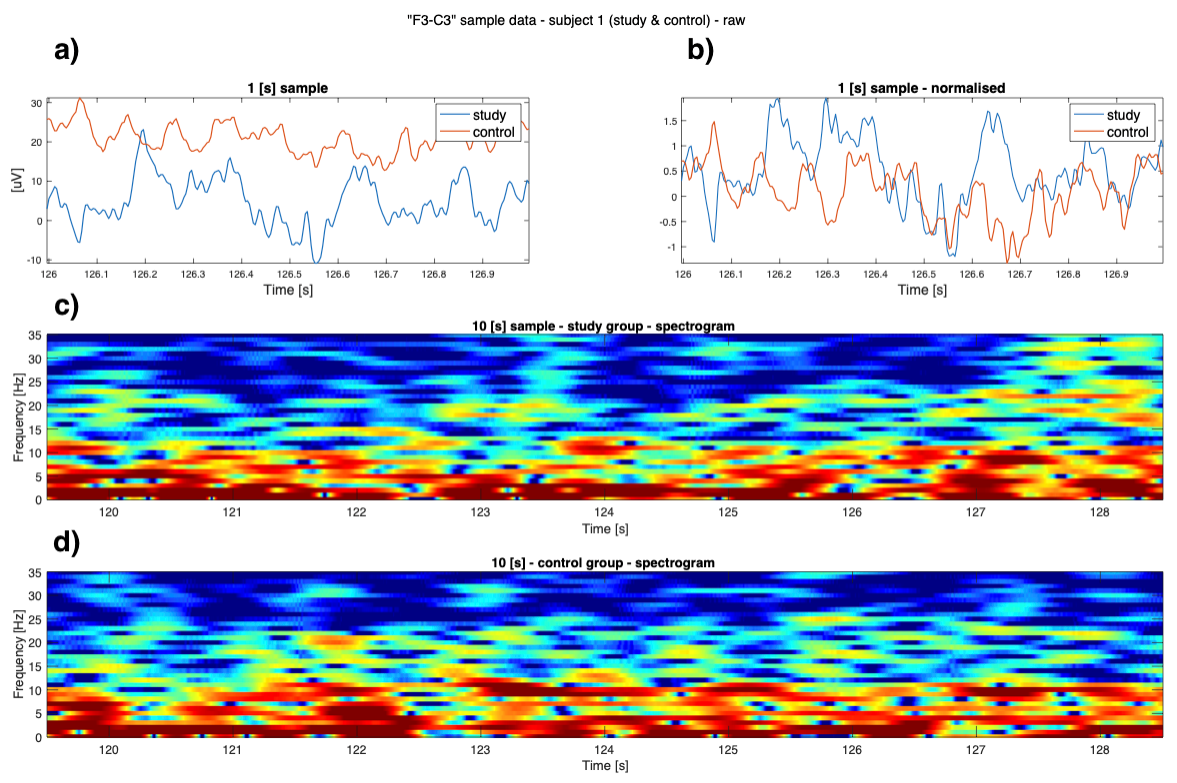
Example raw data from channel **’F3-C3’**: (**a**) time-series of both study group and control group (1 [s]); (**b**) normalised time series of study group and control group (1 [s]); (**c**) 10 [s] sample of FASD (top spectrogram); (**d**) 10 [s] of healthy control (bottom spectrogram)—sample 1.

As can be seen in Figure 1, Figure 2, Figure 3 and Figure 4 the EEG data differ in children affected by FASD from those from the healthy control group–in particular the “alpha” (7–12 Hz) frequency, which can be observed with reduced power, is visible in the data. A previous study has also shown this result [29].

Data is presented in Figure 1, Figure 2, Figure 3 and Figure 4 (from the same subject, but different channels) (FASD—top, healthy control—bottom). The spectrograms indicate differences in the alpha waves’ density. The presented data is unprocessed.

**Figure 5:**
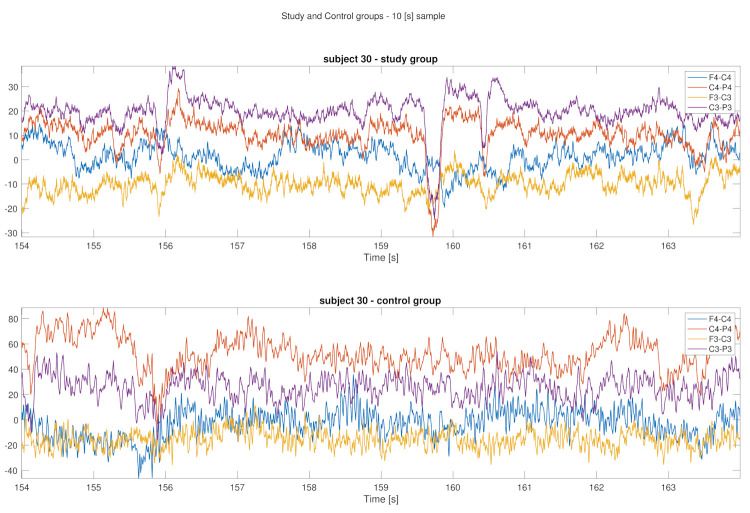
10 [s] sample from four channels (**’F4-C4’**, **’C4-P4’**, **’F3-C3’** and **’C3-P3’**)—study group subject (**top**) and control group subject (**bottom**).

Figure 5 illustrates a 10 [s] long sample recorded from channels: **’F4-C4’**, **’C4-P4’**, **’F3-C3’** and **’C3-P3’**). Samples were from two subjects, one inform the study (top) and one from thecontrol (bottom) group. Some movement can be observed, namely eye-blink-related artifacts in the study group data, which might have been caused by some hyperactivity of the child with FASD [37,38,39].

**Figure 6:**
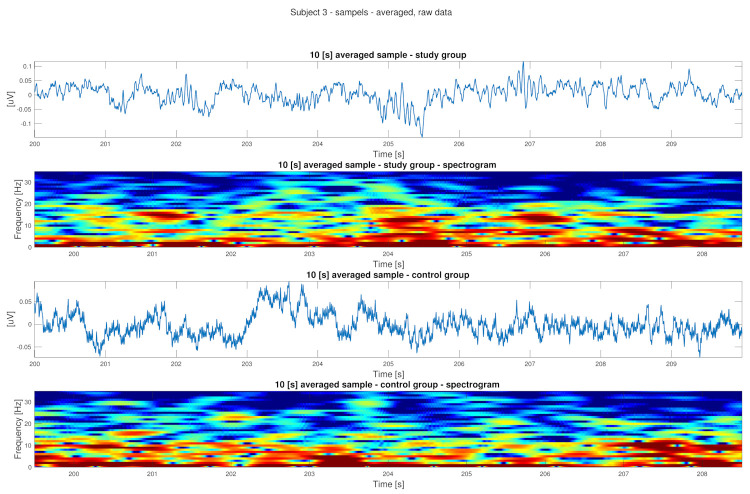
Averaged sample study group (**top**) and control group (**bottom**)—time series and spectrogram (10 [s]).

In Figure 6, a 10 [s] averaged data from two subjects (study—top, control—bottom) is presented.

**Figure 7:**
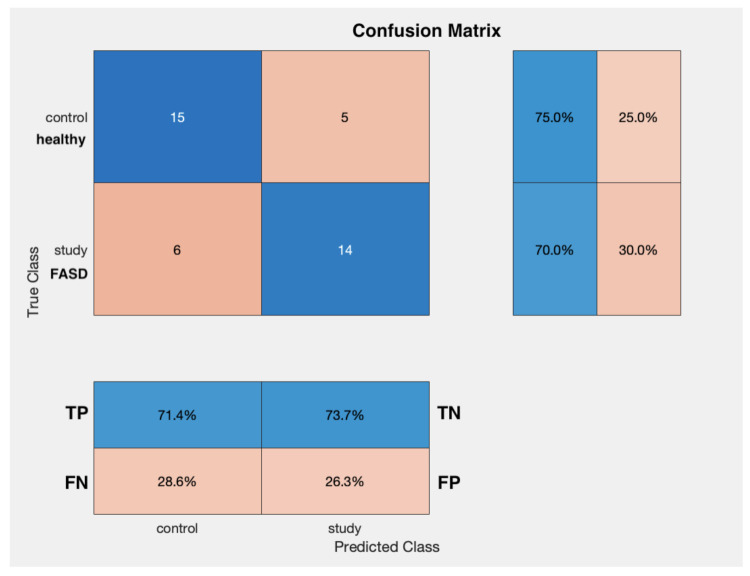
Result of using the Naive Bayesian classifier—confusion matrix.

Figure 7 presents the result of using the proposed naive Bayesian classifiers in this study, the results were presented with the confusion matrix.

TP stands for True Positive and equals 71.4%, where 26.3% was where the signals were falsely recognised as similar (FP—False Positive). In 73.7% cases the signals were recignised as different (TN—True Negative), where 28.6% was recognised as FN (False Negative).

The efficiency of the proposed method is 75%, which is relatively high compared to other studies using naive Bayesian classifiers, but it is important to mention, that no similar studies could be found in the literature. In our study 30 subjects (in each group—control and study) were used to teach the classifier and 20 for testing purposes. As it was mentioned above—the use of naive Bayes classifiers requires only a small amount of training data set, which was one of the reasons for choosing it for this study purposes.

The data was shuffled each time. There were 1000 trials after which the data was shuffled. The accuracy was 78% and SD = 0.03 (3%). The classifier was trained each time with the data that were randomly selected from sets in appropriate proportions. The classifier was learned several thousand times, but the changes in classification accuracy were not significant. We chose the 60–40 split due to the small set of data and their nature. In addition, the Bayesian classifier is a classifier based on stochastic information in the data, which allows it to predict its behavior in the case of determining data characteristics. It was the Bayesian classifier with 4th order k-fold cross-validation.

## 4. Discussion

The direct mechanism in which alcohol affects the neurophysiological activity of the brain still remains unknown. It has been established that alcohol crosses the placental and blood-brain barriers affecting developing neurons of fetal brain [40]. Cell- and animal-related studies have demonstrated that the possible neurobiological mechanisms are based on expanded apoptosis, modified neural migration, altered cell cycle kinetics and neuroinflammation [40]. All these factors are combined into a vicious circle. Alcohol can lead to neurotransmitter changes, particularly GABA [41,42] and NMDA [43,44]. Neurotrophins alterations not only directly cause cell death but also cause changes in the neural plasticity leading to altered trafficking of ion channels [45] and changes of intrinsic excitability [46] which can cause imbalance in excitation and disinhibition, which can cause a cell death. Another causes of apoptosis are oxidative stress-mediated cell deaths [47], neuroinflammation and glia activation [48,49,50]. An increased cell-death processes results in plastic changes of brain structure leading to a decrease of neural network activity [51]. All mentioned pathways and loops have an effect on anatomical structure along with sensory and cognitive functions of alcohol-exposed brains. Many studies, based on magnetic resonance imaging (MRI), have reported that FASD patients brain structures, being mainly the corpus callosum, hippocampus and basal ganglia are altered in the volume and shape [52,53,54]. The cortex is also thinner compared to normal brains [55]. On the other hand, diffusion tensor imaging studies have demonstrated lower fractional anisotropy in the area of corpus callosum [56]. Reductions in connectivity between cortical structures and deep grey matter have been shown in resting-state functional MRI [57], and reduced connection, executive and attention networks were presented in FASD patients [58].

## 5. Conclusions

These initial results indicate that on the basis of the EEG it is possible to detect FASD, even in children and adolescents without visible external features, making it is a promising and efficient tool, whichcan potentially support the diagnostic process. It is non-invasive and inexpensive compared to other medical imagining methods [33,34]. The main problem with the FASD diagnosis is the various applied criteria, which differ across countries and even among clinics themselves. Most of the screening criteria are based mostly on demographic, maternal alcohol consumption and physical features, which are time- and cost-inefficient and which lead to frequent misdiagnosis [7]. A potential alternative is diagnosis based on EEG signals, like the one presented in this work.

This is the first study (based on the literature background) to evaluate the role of EEG analysis with the use of naive Bayesian classifiers in FASD diagnosis. Moreover, we were able to include in the analysis the EEG signals from a significant group of patients with FASD.

### 5.1. Limitations of This Study

There are some limitations of this study that need to be mentioned. Firstly the EEG examinations were performed for the clinical purposes that caused a variability in some study phases (duration of eyes closed, eyes open etc.). The naive Bayesian classifier has been used in order to overcome these limitations. Moreover, the retrospective data acquisition implicated the inability to exclude potential confounders; however, exclusion criteria based on the patients’ history available from the database were applied. Another issue was the problem of gender and age inequality in the study and control groups, caused by the fact that participants were recruited in a clinic on voluntary basis.

Due to the nature of the research and its stage, we did not attempt to compare other classification methods because the amount of data for teaching classifiers such as neural networks on this data set is ineffective due to the amount of data, simpler classifiers, such as e.g., closest neighbors, would be based on metrics which would not take information on the distribution of the data. However, such comparisons are planned in the later stages of the work.

Also the amount of data collected in this study is too small to apply them in neural networks.

### 5.2. Further Research Plans

Further research plans include screening more children and adolescents in order to obtain more data. We also plan to combine the naive Bayesian classifier with some more sophisticated signal processing methods. These are often referred to as hybrid methods, and involve combining two or more signal processing methods. This is the current trend and future in biomedical engineering. Among these methods are blind separation algorithms, such as independent component analysis (ICA) combined with adaptive filtering [34]. The authors are also planning to combine the naive Bayesian classifier with ICA and to customize and improve threshold-based methods, as described in [59].

We are also planning to apply neural networks, like in [60] in the further future (after collecting more data), as our current data set is to small for proper neural networks training.
